# Mechanisms of resistance and susceptibility to experimental visceral leishmaniosis: BALB/c mouse versus syrian hamster model

**DOI:** 10.1186/1297-9716-42-39

**Published:** 2011-02-23

**Authors:** Ana Nieto, Gustavo Domínguez-Bernal, José A Orden, Ricardo De La Fuente, Nadia Madrid-Elena, Javier Carrión

**Affiliations:** 1 Anapath, Anatomic Pathology Laboratory, 18015 Granada, Spain; 2 Department of Animal Health, Faculty of Veterinary, Complutense University of Madrid, 28040 Madrid, Spain; 3 Department of Infectious Diseases, Hospital Ramón y Cajal, 28034 Madrid, Spain

## Abstract

Several animal models have been established to study visceral leishmaniosis (VL), a worldwide vector-borne disease affecting humans and domestic animals that constitutes a serious public health problem. BALB/c mice and Syrian hamsters are the most widely used experimental models. In this paper, we summarize the advantages and disadvantages of these two experimental models and discuss the results obtained using these models in different studies of VL. Studies using the BALB/c mouse model have underscored differences between the liver and spleen in the course of VL, indicating that pathological evaluation of the visceral organs is essential for understanding the immune mechanisms induced by *Leishmania infantum *infection. The main goal of this review is to collate the relevant literature on *Leishmania *pathogenesis into a sequence of events, providing a schematic view of the main components of adaptive and innate immunity in the liver and spleen after experimental infection with *L. infantum or L. donovani*. This review also presents several viewpoints and reflections about some controversial aspects of *Leishmania *research, including the choice of experimental model, route of administration, inoculum size and the relevance of pathology (intimately linked to parasite persistence): a thorough understanding of which is essential for future VL research and the successful development of efficient control strategies for *Leishmania spp*.

## 1. Introduction

The parasitic protozoa of the genus *Leishmania *cause a spectrum of diseases in humans, ranging from subclinical cutaneous infections to more serious disseminating diffuse cutaneous, mucocutaneous and visceral forms of the disease. Leishmaniosis is one of the most prevalent neglected tropical diseases affecting public health worldwide [1,2]. It is transmitted by the bite of female sandflies. In developing countries it is associated with extreme poverty. It is estimated that at least 20 million people are infected with *Leishmania*. The visceral form is the most severe form of the disease. Annually, there are approximately 500 000 new cases of visceral leishmaniosis (VL) [3]. *Leishmania donovani *is the primary cause of VL in the Indian subcontinent and East Africa, *L. infantum *in the areas surrounding the Mediterranean Sea where it is a zoonosis, and *L. chagasi *in the New World. The last two species are identical. Human beings are the only known reservoir of *L. donovani*, while canines provide the reservoir for *L. infantum *and *L. chagasi *[4]. However, since asymptomatic parasitemic injecting drug users who share injecting devices seem to be a suitable reservoir for *L infantum*, an artificial anthro-ponotic cycle would be completed. Needles and syringes would be the vectors and uninfected injecting drug users the receptors [5]. Also, *L. infantum *is known to cause opportunistic infections in patients with HIV/AIDS [6]. *Canis familiaris *is the major host for these parasites, and the main reservoir for human visceral infection [7]. The risk for reintroduction of VL and other vector-borne diseases in Europe as a consequence of global warming has recently been highlighted [8]. Indeed, VL appears not to be limited to the Mediterranean region and has now spread northwards [9].

Manifestations of VL can vary from asymptomatic infection to progressive fatal visceral disease. Disease progression is dependent on both the species of *Leishmania *involved and the genetics and immune status of the host. Active VL is characterized by fever, weight loss, hypergammaglobulinemia, hepatosplenomegaly, anemia, thrombocytopenia, leukopenia and immunodepression [10,11]. Also, the presence of parasite-specific antibodies forming immune complexes in the kidneys may lead to the development of glomerulonephritis [12,13].

Leishmaniosis diagnosis and treatment are expensive. Despite considerable advances, there are still no efficient vaccines available against human leishmaniosis [14,15]. Recently, a vaccine containing the fucose-mannose ligand has been industrialized and licensed for commercialization in Brazilian endemic areas under the name of Leishmune^® ^(Fort Dodge Ltda, São Paulo, Brazil) to prevent canine VL. Unfortunately, the immune response induced by vaccination has not yet been fully investigated. Also, this vaccine is solely recommended for asymptomatic and seronegative dogs [16-19].

**Figure 1:**
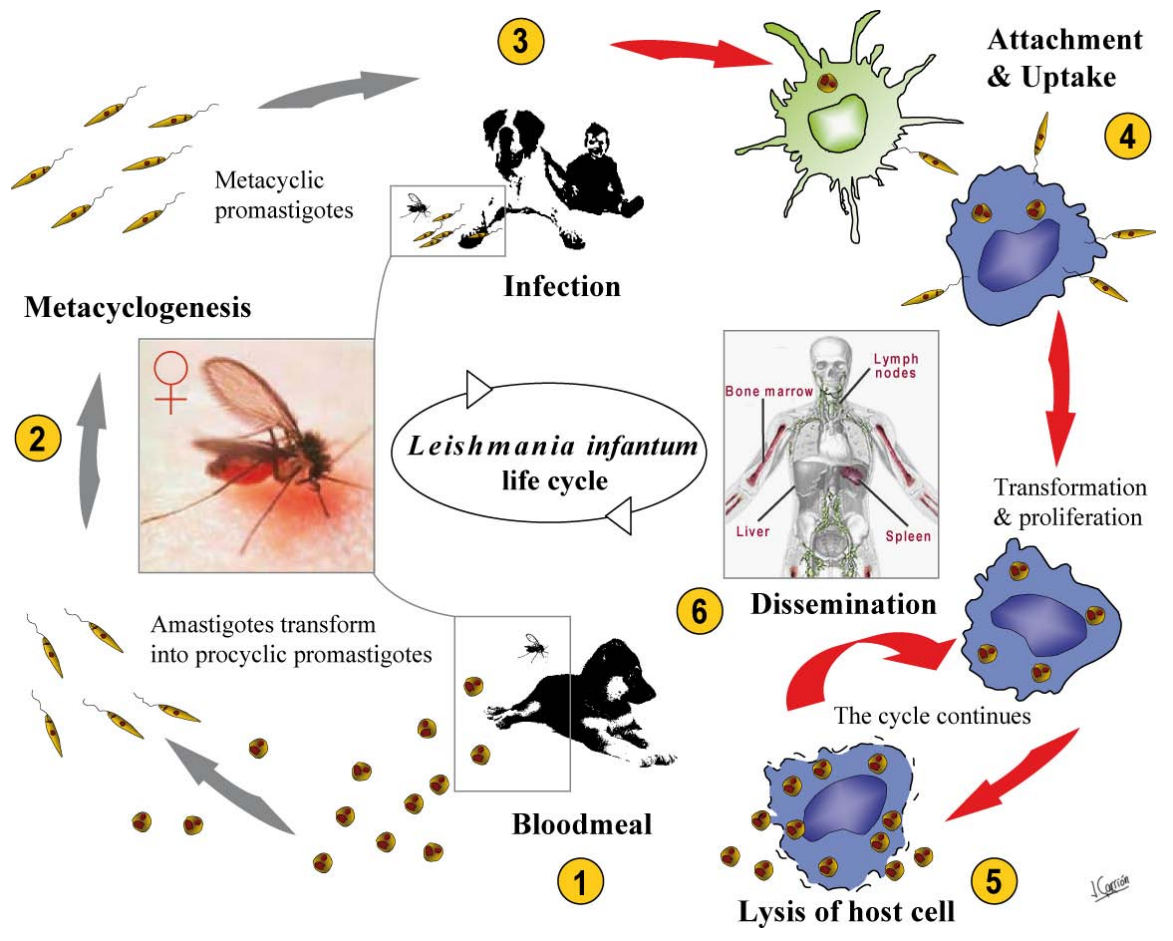
***L. infantum *life cycle**. (1) During the bloodmeal from an infected vertebrate host, the female sandfly ingests free amastigotes, as well as intracellular amastigotes. In the midgut of the sandfly the amastigotes transform into procyclic promastigotes. (2) The procyclic promastigotes multiply and transform into metacyclic promastigotes, the infective stage, that migrate towards the buccal cavity of the sandfly. (3) A bite from the sandfly transmits *Leishmania *promastigotes to susceptible mammalian hosts (i.e., humans and dogs). (4) Promastigotes invade macrophages and DCs. Within these host cells, promastigotes transform into intracellular amastigotes and replicate to produce a large number of parasites. (5) Consequently, the infected cell ruptures and releases amastigotes into the circulation. (6) Free amastigotes can infect other mononuclear phagocytic cells of the blood, spleen, liver, lymph nodes and bone marrow, and the life cycle is repeated.

*L. infantum *has a digenetic life-cycle (Figure 1), alternating between free-living, flagellated, promastigotes in phlebotomine sand flies and obligate, intracellular, aflagellated amastigotes, which preferentially multiply within macrophages or dendritic cells (DCs) of the vertebrate host [20,21].

Several experimental models of VL have been developed, but none of these entirely reproduce the disease in humans [22]. Much of the literature from these models documents the immune parameters contributing to resistance against the visceralizing *Leishmania *species used in vaccine studies [23]. This contrasts with a limited number of studies which have been prompted to study pathological aspects related to VL. In this context, close attention should be given to the histopathological alterations. Humans, dogs and hamsters often exhibit severe clinical signs and symptoms during visceral infection [23-25], whereas mice generally show a few minor signs or no clinical signs at all, depending mainly on the size of the parasite inoculum [26]. Under experimental conditions, progression of visceral disease also depends on the route of infection together with the strain of *Leishmania *parasites used [22]. These factors make the choice of a suitable laboratory model difficult. Studies using experimental murine models of VL do not allow exact extrapolations to be made concerning susceptibility in dogs and humans, but increase the ease of identifying genes and predicting their functional roles, as well as investigating the immune mechanisms involved in human and canine leishmaniosis. This review will aim to provide a better understanding of a variety of pathological-immune responses that have been described to date in the most widely used experimental models of VL (Syrian hamsters and BALB/c mice). Combining research approaches at the immunological, pathological and genetic levels helps to advance our understanding of the mechanisms involved in visceral infection at different stages of the disease.

## 2. Syrian hamster model of VL: suitability of this experimental model

The usual routes of infection in the hamster model of VL are intracardiac and intraperitoneal. However, the administration of parasites by the saphenous vein in order to minimize stress on the hamsters has also been reported [27]. Experimental studies in *L. infantum *and *L. donovani*-infected Syrian hamsters (*Mesocricetus auratus*) often reveal several clinical signs of progressive VL (hypergammaglobulinemia, hepatosplenomegaly, anemia, cachexia and immunodepression) that closely mimic active canine and human disease [22,23,25,28,29]. Surprisingly, there are significant amounts of Th1 cytokines (IFN-γ, IL-2 and TNF-α) in the spleen, but there is little or no IL-4. However, to allow the parasites to multiply, deactivating Th2 cytokines (TGF-β and IL-10) may act on infected macrophages as well as anti-*Leishmania *antibodies (which have no protective role in leishmaniosis) that opsonize amastigotes and induce IL-10 production in macrophages. These high activation and deactivation processes are likely to occur mainly in the spleen and liver [30]. Interestingly, Syrian hamsters exhibit reduced expression of the gene encoding inducible nitric oxide synthase (iNOS) in response to IFN-γ, and this is thought to lead to a low nitric oxide (NO) generation, subsequently defaulting in parasite killing [10,28,31]. Furthermore, there is a lack of reagents for immunological analysis in the hamster model of VL. Taking these factors into account, we consider the Syrian hamster to be a suitable experimental model for the study of the pathological features of active VL (as described below), but it is not a suitable model for the evaluation of immunization strategies, as a result of the animal's high innate susceptibility.

In Syrian hamsters, manifestations of VL can range from asymptomatic and oligosymptomatic infections to progressive fatal visceral disease [28]. The pathological features reported during VL include hypoplasia of the white pulp in the spleen, hepatic granulomas and the deposition of a secondary amyloid substance both in the spleen and the liver [32,33]. Also, other studies of active VL have reported that infected hamsters develop glomerulonephritis associated with deposition of immunoglobulins and parasite antigens (immune complexes) in the kidneys. Finally, the disseminated amyloidosis and glomerulonephritis produce renal failure and nephrotic syndrome in infected hamsters [12,34]. The visceral infection in hamsters also induces pathological alterations in hepatocytes, mainly in the endomembrane system and the peroxisomal compartment, leading to a disturbance of liver metabolism [35]. In a recent study [36], hamsters infected with *L. infantum *were shown to develop analogous inflammatory myopathies to those observed in naturally infected dogs [37]. Taken together, all these factors probably contributed to the immune response disorders that resulted in the death of the animals [22,33].

**Figure 2:**
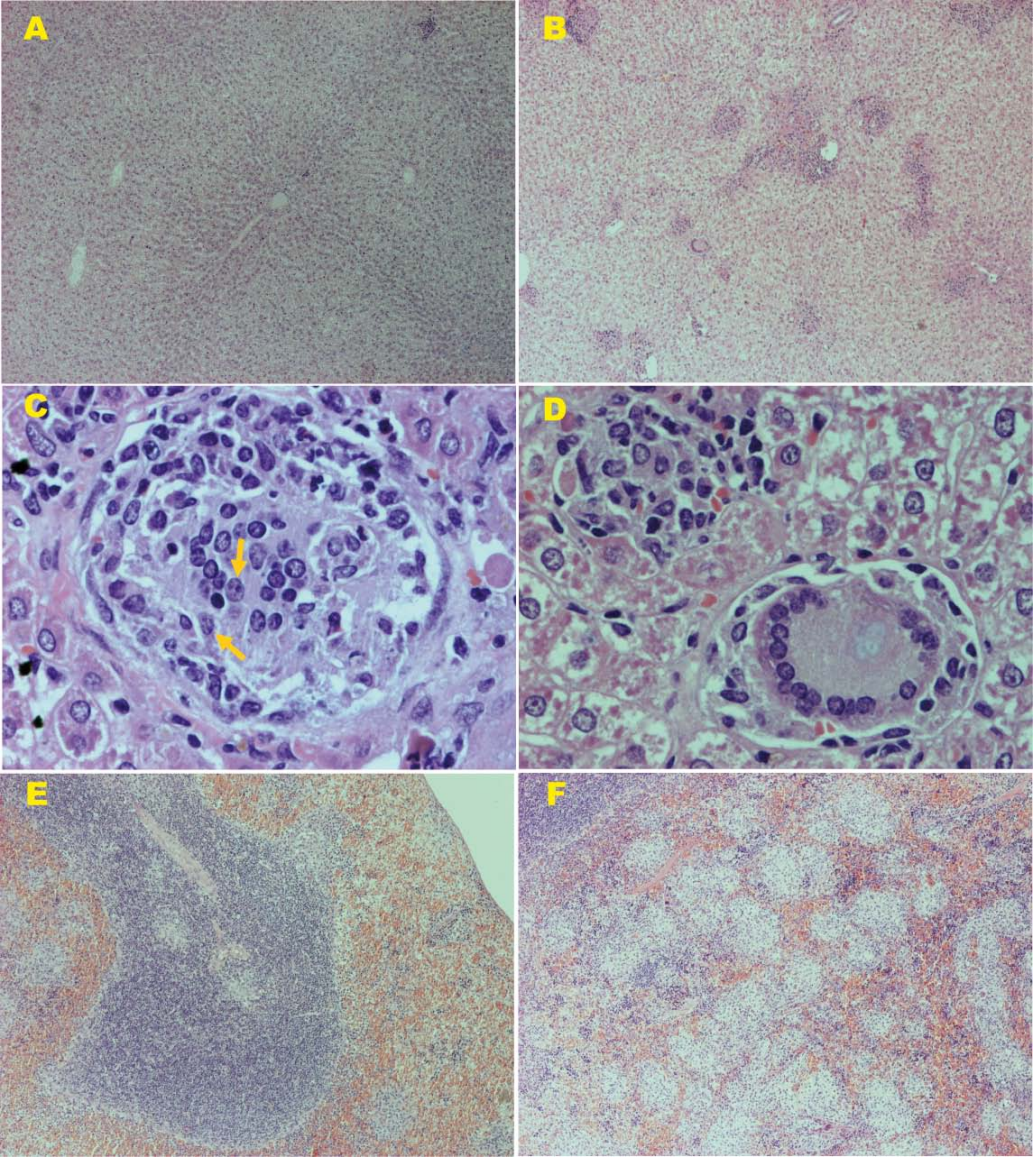
**Liver and spleen histological sections from Syrian hamsters stained with H&E**. (A) Uninfected hamsters show normal liver histological sections (×40). (B) Hamsters infected with 10^5 ^*L. infantum *parasites show granuloma reactions after three months pi (×40). Compare (A) with (B). (C) Granuloma formation. Initial parasitization of KCs (arrows) surrounded by a few inflammatory cells (lymphocytes and monocytes), showing the lack of organization after three months pi (×400). (D) Developing granuloma and giant cells containing few residual amastigotes after three months pi (×400). (E) Normal splenic architecture in control hamsters (×40). (F) Disruption of the splenic architecture accompanied with lymphoid depletion in hamsters infected with 10^7 ^*L. infantum *promastigotes after three months pi (×40). Compare (E) with (F). Hamsters and BALB/c mice were purchased from Harlan Interfauna Ibérica S.A. (Barcelona, Spain). The animals were maintained under conventional conditions approved by the Ethical Committee for the Animal Experimentation of the Complutense University of Madrid.

**Figure 3:**
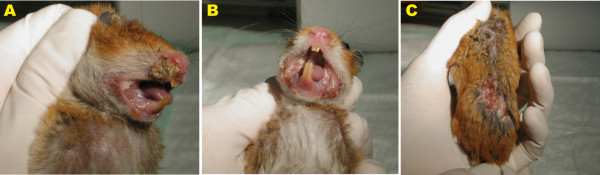
**External lesions observed in Syrian hamsters infected with 10^7 ^*L. infantum *promastigotes at seven months pi**. (A-B) Mucocutaneous lesions localized in the snout. (C) Ulcers on the back of the hamsters. The isolate M/CAN/ES/96/BCN150 (zymodeme MON-1) of *L. infantum *was used for infection experiments. This strain was maintained in our laboratory by passage in Syrian hamsters.

Pathological studies from our laboratory showed that after *L. infantum *intracardiac infection, hamsters exhibited severe histopathological alterations in both the spleen and liver at the peak of parasite burden. Among these alterations, we detected the apparition of granulomas in different maturation stages and giant cell granulomas with amastigotes in the liver (Figure 2A-D), as well as disruption of the splenic architecture accompanied by lymphoid depletion (Figure 2E-F). Interestingly, several months after intracardiac infection with 10^7 ^promastigotes of *L. infantum*, we found external mucocutaneous lesions localized in the snout, accompanied by ulcers on the back of the animals (Figure 3).

## 3. Mouse model of VL: genetic control of susceptibility to *L. infantum *infection

Genetic control studies of various host defense mechanisms in the mouse (*Mus musculus*) model during the course of progressive infection with visceralizing *Leishmania spp*. are summarized in Table 1. These experiments made an important contribution in identifying genes involved in VL innate and acquired immunity. Identification of the *Slc11a1 *gene aided our understanding of the susceptibility at early stages of infection in BALB/c mice, which reflects the strength of the innate immune response in controlling early parasite growth independently of acquired immune mechanisms. The *Slc11a *gene also controls susceptibility to bacteria. Indeed, mutations in *Slc11a1 *cause susceptibility to infection with *Salmonella spp*. [38] and *Mycobacteria spp*. [39]. Interestingly, iron is required for replication of pathogens such as *Leishmania *parasites in phagosomes. The *Slc11a1 *gene encodes a protein expressed on the membrane of infected phagosomes that removes Fe^2+ ^and Mn^2+ ^ions from the intra-phagosomal compartment restricting intracellular *Leishmania *multiplication in iron-limited intracellular environments [40,41]. Genetically resistant mouse strains (e.g., CBA) possess a functional *Slc11a1 *gene which confers innate resistance to early *Leishmania *parasite growth. In contrast, susceptible mice strains (e.g., C57BL/6 and BALB/c) possess a non-functional *Slc11a1 *gene and early parasite growth in the liver cannot be controlled [42]. However, most susceptible mouse strains, including BALB/c, develop acquired immune mechanisms to control hepatic parasite growth at later stages of infection (as previously reviewed [43,44]).

**Table 1:** Genes that control the immune response to *L. donovani/L. infantum *infection.

Host defense mechanism(s)	Locus or gene	Chromosome	Reference(s)
Innate intraphagosomal control of infection in the spleen and the liver	*Slc11a1*	1	[40-44]

Influences antigen presentation during the acquired immune response in the splen, the liver and the bone marrow	*H2*	17	[43,45]

Formation of hepatic granulomas. Acquired immune response	*Ir2*	2	[43]

Influences resistance to parasites in the spleen. C57BL/6J bg/bg mice expressed deficient natural killer cell activity and failed to eliminate *L. donovani *amastigotes	*Lyst/Beige*	13	[46]

The parasite load in the liver at later stages of infection, which probably reflects the strength of the acquired immune response, was found to be controlled by the *H2 *and *Ir2 *loci. The haplotype at the *H2 *genomic region on chromosome 17 is involved in antigen presentation through the major histocompatibility complex (MHC). Genetic polymorphism in the MHC influences the response to numerous antigens. Several MHC haplotypes have not only been associated with resistance to leishmaniosis, but also with resistance to many other infections [45]. Differences between the *H-2^b ^*and *H-2^d ^*haplotypes were observed in the BALB/c background, where *H-2^b ^*resulted in lower parasite numbers in the liver than *H-2^d^*. In addition to the liver, the *H2 *region influences parasite numbers in the spleen and bone marrow [43]. Histopathological analysis revealed that the *Ir2 *locus in mice promoted fewer granulomas that were smaller in size, due to an efficient anti-parasite response.

The parasite burden in the spleen was also found to be controlled by the *Lyst/Beige *gene on chromosome 13. Indeed, homozygous C57BL/6J bg/bg (beige) mice expressed deficient natural killer (NK) cell activity and failed to eliminate *L. donovani *amastigotes [46].

## 4. BALB/c mouse model of VL: organ-specific immune responses

**Figure 4:**
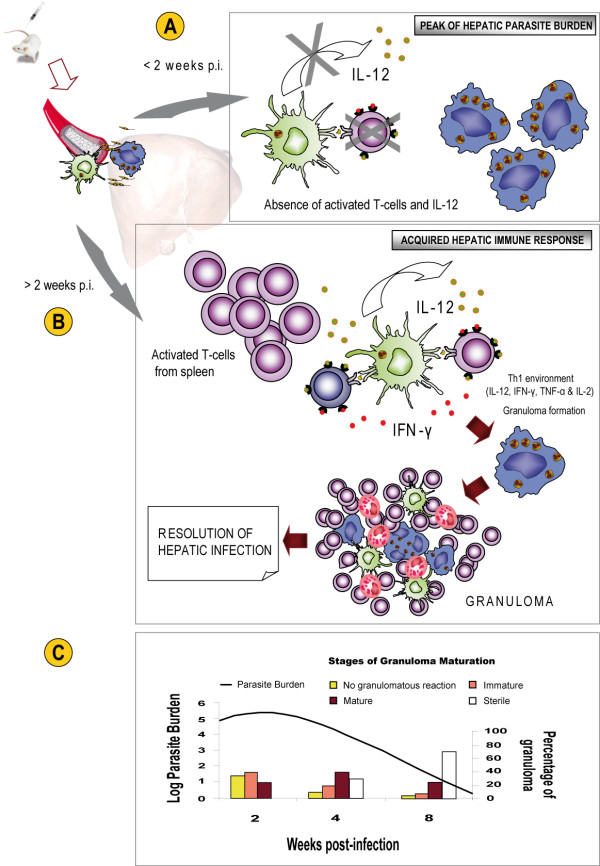
**A schematic view of the immune responses in BALB/c mice livers after experimental infection with *L. infantum***. After intravenous inoculation, the parasites enter the liver and invade macrophages and DCs. (A) During the hepatic acute phase (up to two weeks pi), *Leishmania *amastigotes multiply in the absence of both IL-12 production and activated T-cells. Consequently, the number of parasites in the liver reaches a peak. (B) Two weeks pi, *Leishmania*-specific T lymphocytes migrate to the liver from the spleen and the acquired hepatic immune response is initiated. The interaction of *Leishmania*-specific T cells with infected KCs and DCs provides the proinflammatory (Th1) environment required for efficient granuloma formation, resulting in the resolution of hepatic infection. (C) The kinetics of parasite burden and different stages of granuloma maturation in the liver after *L. infantum *infection. Infected KCs with no granulomatous reaction and immature granulomas were observed in high numbers at 14 days pi but these initial stages of granuloma formation decreased in number during the course of infection, developing mature and sterile granulomas. Significantly, by 56 days pi, the number of sterile granulomas, in which the amastigotes were killed, increased and consequently, the *Leishmania *parasite burden decreased. Finally, the infection in the liver of BALB/c mice was resolved.

The variations in the susceptibility to VL in different strains of mice were first described nearly 40 years ago [47]. In BALB/c mice, the immune response to *L. infantum *and *L. donovani *infection can vary markedly between different organs (liver and spleen) within the same animal. In the liver, the infection can resolve with subsequent immunity to re-infection, whereas in the spleen, *Leishmania *parasites can persist [48]. A schematic view of the organ-specific immune responses after experimental infection with *L. infantum *in susceptible BALB/c mice is shown in Figures 4 and 6.

**Figure 5:**
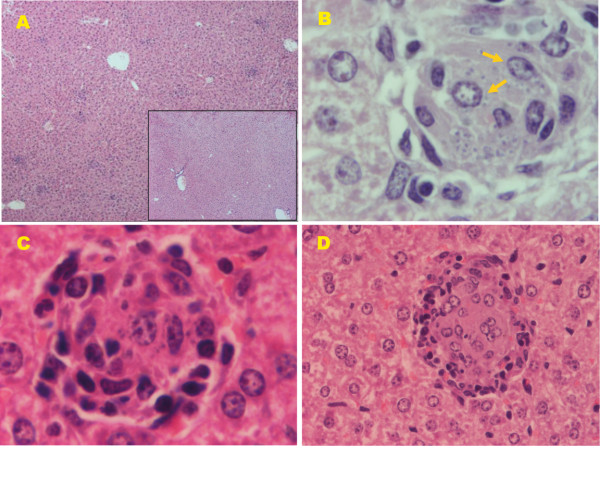
**The liver granuloma reaction after *L. infantum *infection**. (A) The histology of the liver and cellular infiltrates in infected mice at 28 days pi (×40). Compare (A) with the detail in the lower right corner of the image (non-infected mice showing normal histological appearance, ×40). Liver granuloma evolution: (B) immature granulomas. Early parasitization of KCs (arrows) with initial cell recruitment (×400), at 14 days pi. (C) Mature granuloma assembly at infected KCs, resulting in the attraction of lymphocytes and monocytes, at 28 days pi (×400). (D) Mature-sterile granuloma, free of amastigotes, at 56 days pi (×200).

**Figure 6:**
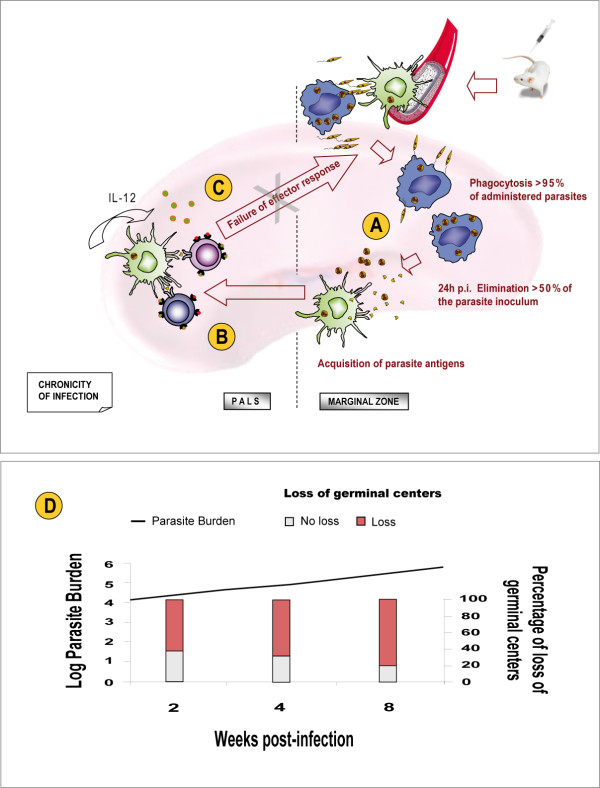
**A schematic view of the immune responses in the BALB/c mice spleens after experimental infection with *L. infantum***. (A) In the initial stage of infection, parasites from the blood invade macrophages and DCs into the splenic MZ. Also, DCs acquire parasite antigens in the MZ and subsequently migrate to the PALS. (B) DCs produce IL-12 and present parasite antigens to T cells in the PALS. (C) *Leishmania*-specific T-cells are activated in the PALS but a failure in the specific effector response prevents them from interacting with parasitized host cells in the MZ. Finally, chronicity of infection occurs in the spleen. (D) The kinetics of parasite burden and loss of germinal centers in the spleen after *L. infantum *infection. The progressive loss of splenic germinal centers increased with time. Thus, in the spleen, a site of chronic infection, the high levels of depletion in the white pulp at 56 days pi correlated with the high *Leishmania *parasite burden.

### 4.1. Liver: control of hepatic infection

#### 4.1.1. Development of an immune response to the early stage of infection

After being inoculated into the lateral vein of the tail, the parasites enter the liver via the portal vein and invade macrophages and DCs. In both these types of host cell, promastigotes transform into amastigotes. At this point, the innate immune system constitutes its first line of defence against *Leishmania *parasites. The parasitized resident macrophages (Kupffer cells, KCs) secrete chemokines (CCL3, CCL2 and CXCL10) that stimulate the recruitment of monocytes and granulocytes [44]. Despite the activation of these mechanisms in mice, amastigotes survive during the hepatic acute phase (up to two weeks post-infection (pi)) in an environment with small quantities of inflammatory cytokines, in the absence of activated T cells. Consequently, the number of parasites in the liver reaches a peak (Figure 4A). Nevertheless, the parasite burden may decrease dramatically with the acquisition of the granulomatous response during the next stage of infection, as described below.

#### 4.1.2. Development of an immune response to the later stage of infection: granuloma formation

Recent research favours a model in which *Leishmania*-specific T lymphocytes are pre-activated in the spleen and then migrate to the liver [48]. Once there, activated T cells interact with parasitized DCs, serving as a critical source of IL-12 production, that then triggers the subsequent *Leishmania*-specific CD4^+ ^Th1 effector response during the later stage of infection [21]. Interestingly, activated DCs can also trigger NK cell cytotoxicity and the production of IFN-γ [49]. In contrast to DCs, the production of IL-12 is blocked in infected macrophages. Consequently, the parasite-carrying macrophages are incompetent at priming CD4^+ ^T cells or stimulating antigen-specific CD4^+ ^T cells [50]. Therefore, the interaction of *Leishmania*-specific CD4^+ ^T cells with infected DCs in the liver provides the proinflammatory (Th1) environment required for efficient granuloma formation (Figure 4B), which includes IL-12, IFN-γ, TNF-α and IL-2 production [10,48,51-53]. It is at this stage of infection (weeks 2-4 pi) that the acquired hepatic immune response is initiated. Simultaneously, the fusion of infected KCs to form multinucleated cells also contributes to inflammatory cytokine production during granuloma formation [54,55]. In BALB/c mice, acquired hepatic resistance to *L. infantum *clearly depends upon granuloma development. Thus, the structure of a mature tissue granuloma consists of a core of fused, parasitized KCs with an encircling mononuclear cell mantle containing blood monocytes and both CD4^+ ^and CD8^+ ^T cells. In some instances, B cells, plasma cells and granulocytes are also attracted. In immunologically active granulomas, antigen-presenting DCs and cytokine-secreting T cells are required for antimicrobial activity [54]. The formation of a granuloma is not always associated with parasite control, and the effectiveness of hepatic granulomas to kill parasites depends on their degree of maturation [52,54]. It appears that the TNF family of cytokines are not involved in the formation of granulomas but instead are involved in their maturation, as well as the maintenance of splenic architecture [42].

Granulomas become fully evolved by 2-4 weeks pi. The overall antimicrobial efficacy of the granulomatous response appears to be variable, and only mature granulomas develop efficient leishmanicidal mechanisms to kill parasites. In contrast, developing granulomas have been reported to be less efficient at killing *Leishmania *parasites. Among other factors, granuloma development has been found to vary depending on the initial inoculum size. Indeed, higher numbers of mature and sterile granulomas are observed in mice infected with a low-inoculum size than in those infected with a high-inoculum size [26]. In structurally mature hepatic granulomas, the elaboration of leishmanicidal reactive oxygen intermediates (ROIs) and reactive nitrogen intermediates (RNIs) is essential for parasite killing within infected KCs and DCs [44,54].

There are various classification schemes for granulomatous inflammation in VL. Murray et al. [54] reported a summary of liver granuloma structure-function relationships in experimental VL. To score the progression of the granulomatous response, Stager et al. [56] also classified the infected focus as follows: (1) an infected KC with no associated cellular infiltrate, (2) an early granuloma comprising an infected KC surrounded by a few inflammatory cells, with no organization, (3) a mature granuloma with an organized structure, or (4) a sterile granuloma, in which amastigotes had been killed as a result of effective antileishmanial immunity. Following the above criteria our laboratory data also revealed that the resolution of disease in the livers of mice infected with *L. infantum *correlates with granuloma development (Figure 4C and Figure 5A). Early in the course of infection, granulomas at various stages of maturation are apparent [44]. Thus, relatively mature granulomas can be readily detected alongside infected KCs that have no associated cellular infiltrate at around four weeks pi (Figure 4C). Infected KCs exhibiting no granulomatous reaction and immature granulomas (Figure 5B) were observed in high numbers at two weeks pi, but their numbers decreased during the course of infection as mature (Figure 5C), sterile (Figure 5D) granulomas developed, in which the amastigotes were killed. After eight weeks pi, sterile granulomas gradually dissembled in an involution process [54]. Although sterile cure is never achieved in the liver, parasite growth is controlled without inducing pathology and it is resistant to secondary infections with *L. infantum *[44]. It is possible that parasite persistence might mediate long-term immunity in the liver in a similar manner to that seen in the cutaneous leishmaniosis model caused by low-dose infection with *L. major *[57].

Studying the granulomatous response is important because granuloma development has been associated with *Leishmania *infection in the liver, as demonstrated in rodent models. Moreover, enhanced granuloma maturation represents a good marker of successful vaccination against VL [58].

### 4.2. Spleen: visceralizing *Leishmania *parasites persist and destroy the splenic architecture

In contrast to the liver, the spleen and bone marrow become chronically infected in mice [44]. The immune response to *L. infantum *in the spleen (Figure 6A-C) can be separated into two phases: acute and chronic.

#### 4.2.1. The acute phase of infection

Following intravenous experimental infection in mice, *L. infantum *promastigotes enter the spleen via the splenic artery and are rapidly removed from the circulation in the spleen by marginal zone (MZ) macrophages and rarely by DCs. It is likely that the majority of DCs acquire *Leishmania *antigens by phagocytosis of infected macrophages or their remnants in the MZ [44,59]. Within these cells, promastigotes replicate intracellularly as amastigotes. In the spleen, MZ macrophages phagocytose > 95% of intravenously administered *L. infantum *parasites, where > 50% of the initial parasite inoculum is killed within 24 h of infection [48]. It appears that DCs acquire parasite antigens within the MZ (Figure 6A) and subsequently migrate to the periarteriolar lymphoid sheath (PALS). Once in the PALS, DCs secrete IL-12 [60] and present parasite antigens to T and NK cells, resulting in the activation of these effector cells (Figure 6B). Interestingly, *L. infantum *infection stimulates IL-12 production by splenic DCs within the PALS, but not infected macrophages within the MZ [61]. As described above, evidence suggests that *Leishmania*-specific T lymphocytes are primed in the spleen during the acute stage of infection (< 4 weeks) and then migrate to the liver to initiate a granulomatous response [42,44,48,62].

#### 4.2.2. The chronic phase of infection

During the chronic stage of infection (> 4 weeks) in the spleen, failure to resolve *L. infantum *infection occurs (Figure 6C) and the splenic architecture breaks down. There are at least three possible explanations for the failure of the specific effector response [48]: (1) assuming that the priming of T and NK cells by DCs occurs at the PALS, a site that is anatomically segregated from the MZ, infected macrophages fail to produce chemoattractants to bring effector cells into their vicinity. (2) Infected macrophages are unable to activate intrinsic leishmanicidal mechanisms following exposure to cytokines and ligands from T and NK cells. It has been reported that *L. infantum*-infected macrophages fail to produce IL-12 and also have a reduced capacity to generate both ROIs and NO, which are important microbicidal molecules for killing intracellular pathogens [44,54,61]. (3) Failure in the development of the efficient granulomatous immune effector response occurs in the spleen. Together, the low expression levels of MHC class II on *L. infantum *macrophages and their intrinsic defects in the generation of an antileishmanial response (see above), contribute to failure to form inflammatory foci around infected MZ macrophages [48].

Any of these three possibilities may contribute to the failure of the spleen to resolve murine VL. Paradoxically, the spleen is an initial site for the generation of cell-mediated immune responses, but ultimately becomes a site of parasite persistence, with associated immunopathological changes [44].

#### 4.2.3. Pathological changes in the spleen

**Figure 7:**
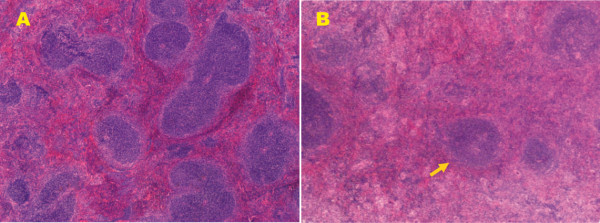
**Changes in splenic anatomy after *L. infantum *infection**. (A) Non-infected mice showed normal histological appearance (×40). (B) After 56 days pi, the splenic architecture showed a significant loss of germinal centers (arrow) in the white pulp (×40). Compare (A) with (B).

In the spleen, *L. infantum *parasite persistence is accompanied by a failure of granuloma formation, splenomegaly and other pathological changes, such as the disruption of splenic microarchitecture, including the disintegration of the white pulp accompanied by the destruction of follicular DCs, and the absence of germinal centres [10,22,24,42,48,61,63]. Interestingly, there is evidence that high levels of TNF mediate the destruction of MZ macrophages, while IL-10 promotes impaired DC migration into T-cell areas with subsequent ineffective T-cell priming [44]. Data from our laboratory showed that during the acute stage of infection (< 4 weeks), parasite burden and the level of splenic disruption increases with time (Figure 6D). Previously, we have reported that the intensity of lymphoid depletion can vary depending on the initial inoculum size. Indeed, higher numbers of lymphoid-depleted BALB/c mice were observed when a high-inoculum size was used compared with a low-inoculum size [26]. In agreement with previous studies [44], our findings revealed the progressive development of splenic pathology in mice infected with *L. infantum*, including disruption of tissue anatomy accompanied by the loss of germinal centers (Figure 7).

## 5. Remarks and discussion

The experimental murine model of *L. infantum *infection mimics many of the features of canine and human infections. Syrian hamsters also exhibit severe clinical signs and symptoms that are similar to those observed in naturally infected dogs and humans [23,24,36]. However, the absence of iNOS expression [10,28,31] and the suppression of lymphoproliferative responses [64,65] observed, lead us to argue that the hamster is a more suitable model for pathological studies of VL than for the evaluation of vaccine candidates. However, to date, most researchers have elected to use the BALB/c mouse model for investigating disease pathogenesis of VL, as well as for vaccine studies [26,44,54,66-68]. In mice, the susceptibility to visceralizing *Leishmania *species is mainly determined by the *Slc11a1 *gene that encodes a phagosomal component that confers the ability to control the early infection (as described above). Even so, BALB/c mice, lacking this gene, are able to control the infection at a later stage [42,69]. In this context, BALB/c mice provide a better model of self-healing or subclinical infection than of disseminated visceral disease [10]. Paciello et al. [36] reported that susceptible mouse strains do not reproduce progressive disease as observed in human active VL. Furthermore, the intensity of pathological changes in the visceral organs of BALB/c mice can vary depending on the initial inoculum size, as described above. Indeed, we proposed that infecting mice with a large inoculum constitutes a suitable model for the study of the pathological changes of VL [26].

Infection with *L. infantum*, either intravenously or intradermally, leads to organ-specific immune responses that are important determinants of disease outcome in BALB/c mice [10,48]. Apparently, the intravenous route of inoculation does not mimic natural infection by the sandfly [22]. However, during natural infection, the blood-sucking action of the vector on the skin of the host may result in both intravenous and intradermal administration of the parasite [26]. Subsequently, parasites multiply rapidly for the first few weeks in the liver. Curiously, the spleen is the initial site of generation of specific T effector cells with the ability to move to the liver. Once in the liver, the development of cell-mediated immune responses is essential for the clearance of *L. infantum *parasites. In contrast, the spleen ultimately becomes the site of parasite persistence [26,44,66,70], suggesting that the spleen is more susceptible to *L. infantum *infection than the liver [71]. Interestingly, the leishmanicidal efficacy of hepatic granulomas is dependent on their degree of maturation [26,51,56]. Therefore, determining the degree of maturation of hepatic granulomas constitutes an effective tool for selecting VL vaccine candidates and for monitoring disease progression.

In summary, it is reasonable to suppose that understanding the development of acquired parasite-specific immunity in the liver and the reasons for effector splenic response failure in VL, may lead to the development of effective strategies for parasite clearance in host target organs during VL and new treatments for canine and human leishmaniosis.

## 6. Competing interests

The authors declare that they have no competing interests.

## 7. Authors' contributions

AN performed the histopathological analyses and participated in the design of the study. GDB, JAO, RDF and NME participated in the design and the discussion section of this paper. JC conceived of the study, carried out the experiments and wrote the paper. All authors read and approved the final manuscript.
